# Identification and Computational Analysis of *BRCA2* Variants in Mexican Women from Jalisco, Mexico, with Breast and Ovarian Cancer

**DOI:** 10.3390/medsci13040248

**Published:** 2025-10-29

**Authors:** Patricia Montserrat García-Verdín, José Elías García-Ortiz, Asbiel Felipe Garibaldi-Ríos, Ingrid Patricia Dávalos-Rodríguez, Sandra del Carmen Mendoza-Ruvalcaba, María Teresa Magaña-Torres, Luis E. Figuera, Mónica Alejandra Rosales-Reynoso, Cesar de Jesús Tovar-Jácome, Guillermo Moisés Zúñiga-González, Belinda Claudia Gómez-Meda, Blanca Miriam Torres-Mendoza, Raquel Villegas-Pacheco, René Gómez-Cerda, Julio César Cárdenas Valdez, Sergio Osvaldo Meza-Chavolla, Martha Patricia Gallegos-Arreola

**Affiliations:** 1 División de Genética, Centro de Investigación Biomédica de Occidente (CIBO), Centro Médico Nacional de Occidente (CMNO), Instituto Mexicano del Seguro Social (IMSS), Guadalajara 44340, Jalisco, Mexico; patricia.garcia@alumnos.udg.mx (P.M.G.-V.); jose.garciaor@imss.gob.mx (J.E.G.-O.); asbiel.garibaldi4757@alumnos.udg.mx (A.F.G.-R.); ingriddavalos@hotmail.com (I.P.D.-R.); qfbmendosan@outlook.com (S.d.C.M.-R.); maganamt@gmail.com (M.T.M.-T.); luisfiguera@yahoo.com (L.E.F.); 2 Doctorado en Genética Humana, Centro Universitario de Ciencias de la Salud (CUCS), Universidad de Guadalajara (UdeG), Guadalajara 44340, Jalisco, Mexico; cesar.tovar4759@alumnos.udg.mx; 3 División de Medicina Molecular, Centro de Investigación Biomédica de Occidente (CIBO), Centro Médico Nacional de Occidente (CMNO), Instituto Mexicano del Seguro Social (IMSS), Guadalajara 44340, Jalisco, Mexico; mareynoso@hotmail.com (M.A.R.-R.); mutagenesis95@hotmail.com (G.M.Z.-G.); 4 Instituto de Genética Humana “Dr. Enrique Corona Rivera”, Departamento de Biología Moleculary Genómica, Centro Universitario de Ciencias de la Salud (CUCS), Universidad de Guadalajara (UdeG), Guadalajara 44340, Jalisco, Mexico; belinda.gomez@academicos.udg.mx; 5 Laboratorio de Inmunodeficiencias Humanas y Retrovirus, División de Neurociencias, Centro de Investigación Biomédica de Occidente (CIBO), Centro Médico Nacional de Occidente (CMNO), Instituto Mexicano del Seguro Social (IMSS), Guadalajara 44340, Jalisco, Mexico; blanca.torresm@imss.gob.mx; 6 Departamento de Disciplinas Filosófico Metodológicas, Centro Universitario de Ciencias de la Salud (CUCS), Universidad de Guadalajara (UdeG), Guadalajara 44340, Jalisco, Mexico; 7 Unidad Médica de Alta Especialidad, Hospital de Gineco-Obstetricia, Centro Médico Nacional de Occidente (CMNO), Instituto Mexicano del Seguro Social (IMSS), Guadalajara 44340, Jalisco, Mexico; villegas.raquel@icloud.com (R.V.-P.); rene1rad@gmail.com (R.G.-C.); julio.cardenasv@imss.gob.mx (J.C.C.V.); 8 Unidad de Detección y Diagnóstico Clínica de Mama, Instituto Mexicano del Seguro Social (IMSS), Guadalajara 44340, Jalisco, Mexico; sergio.mezac@imss.gob.mx

**Keywords:** *BRCA2* gene, *BRCA2* pathogenic variants, breast cancer, ovarian cancer, genetic variation, computational biology

## Abstract

Background: Breast and ovarian cancers (BC and OC) are prevalent malignancies in women globally, with germline variants in the *BRCA2* gene significantly increasing the risk of developing these cancers. Despite extensive studies, the frequency and impact of *BRCA2* variants in women from Jalisco, Mexico, remain underexplored. Objective: The aim of this study was to identify and characterize *BRCA2* gene variants in Mexican women diagnosed with BC and OC and to assess their functional and structural consequences using computational analyses. Methodology: Genomic DNA from 140 Mexican women with BC and/or OC, selected based on clinical criteria suggestive of BRCA2 variants, was sequenced using NGS targeting BRCA2 coding regions. Functional effects were predicted with Ensembl VEP, SIFT, and PolyPhen-2. Structural impacts of missense variants were assessed using HOPE and AlphaFold models. Results: *BRCA2* variants were identified in 12.86% of patients, with higher frequency in OC (21.05%) than BC (12%). Several mapped to key functional domains, including BRC repeats and the DNA-binding domain. Many were predicted as deleterious or probably damaging, though clinical classifications were often conflicting. Structural analysis indicated potential disruptions in protein stability or interactions for most missense variants. Clinically, BRCA2-positive BC patients were younger at diagnosis and showed a trend toward lower complete response. Conclusion: *BRCA2* variants were found in 12.86% of patients, including six VUSs not reported in other populations. Several affected key functional domains with predicted deleterious effects. Findings support the need for genetic panels tailored to the Mexican population.

## 1. Introduction

Breast and ovarian cancer (BC and OC) are among the most common and deadly malignancies affecting women worldwide [1]. Both environmental and genetic factors influence their development. Notably, carriers of germline variants in the *BRCA2* gene have a 55% increased risk of developing BC compared to non-carriers [2]. Furthermore, *BRCA2* variant carriers face a lifetime risk of OC estimated between 11% and 27%, significantly higher than the general population [3,4]. Variants in *BRCA2* can also increase the risk of developing prostate, pancreatic, and colorectal cancers [5,6,7].

The *BRCA2* gene, located on chromosome 13q12.3, plays a crucial role in maintaining genomic stability by regulating DNA repair and cell cycle progression. Its primary function is the repair of DNA double-strand breaks (DSBs) through homologous recombination (HR), a high-fidelity repair pathway essential for maintaining the genome. The BRCA2 protein mediates the recruitment and loading of RAD51 recombinase onto single-stranded DNA at sites of damage, a critical step for the formation and stabilization of the RAD51 nucleoprotein filament. This process involves specific protein domains, including the eight BRC repeats that directly bind RAD51, and the C-terminal domain that further stabilizes the RAD51–DNA complex. BRCA2 also contains a nuclear localization signal (NLS) that ensures proper transport into the nucleus, where DNA repair occurs. Pathogenic variants in *BRCA2* can disrupt these interactions, impairing RAD51 filament assembly, compromising HR efficiency, and ultimately leading to genomic instability and increased cancer predisposition [8,9].

Genetic sequencing has enabled the identification of numerous germline variants in the *BRCA2* gene, classified as benign, probably benign, of uncertain significance, probably pathogenic, and pathogenic [10,11]. However, many variants described in the ClinVar database have conflicting classifications, as their clinical relevance lacks clear consensus among different laboratories, databases, or scientific studies. Large international consortia, such as CIMBA [12] and ENIGMA [13], have conducted extensive analyses linking specific *BRCA2* variants to the risk of BC and OC, providing strong evidence for the pathogenicity of certain variants through case–control studies, segregation analyses, and functional assays. Functional studies have further demonstrated that pathogenic *BRCA2* variants can disrupt RAD51 binding, impair homologous recombination efficiency, and lead to genomic instability [14]. The integration of bioinformatic tools to evaluate the impact of variants on protein stability, domain integrity, and molecular interactions provides valuable complementary evidence to refine variant classification and assess their functional consequences [15].

Although studies describing the frequency and spectrum of clinically relevant *BRCA2* variants have been conducted in different regions of Mexico [16,17,18], none have specifically focused on the population from the state of Jalisco. This regional focus is relevant because Mexico is characterized by marked genetic heterogeneity and admixture patterns that vary by state [19]; for example, Jalisco exhibits a distinctive profile with roughly balanced European and Native American ancestry [20]. In addition, disparities in healthcare access and cancer diagnostic services across the country, including documented delays in rural areas of Jalisco [21], may influence both variant detection and clinical outcomes. These factors underscore the importance of a population-specific analysis to better capture the genetic and clinical landscape of *BRCA2* in this region.

Furthermore, most previous reports have not incorporated computational analyses to predict the functional effects of these variants on the gene or protein, particularly in the case of VUS. Therefore, analyzing this population using both genetic and in silico approaches provides valuable insights into the genetic diversity within the country and may reveal clinically relevant variants not previously characterized in other Mexican cohorts.

In this study, we analyzed *BRCA2* gene variants identified in Mexican women with BC and OC. Our focus was to characterize the frequency of these variants within the Mexican BC and OC population and to perform computational analyses assessing their functional significance, structural stability, and potential impact on the BRCA2 protein.

## 2. Materials and Methods

### 2.1. Patients

This study included Mexican women over 18 years of age with a confirmed diagnosis of BC and/or OC, with clinical suspicion of being carriers of germline pathogenic variants in the *BRCA2* gene, from the population of Jalisco, Mexico. Patient selection comprised women diagnosed with BC and/or OC who met at least one of the following criteria: early disease onset (diagnosis before 50 years of age), a significant family history of related cancers (such as breast, ovarian, prostate, or pancreatic cancer in first- or second-degree relatives), or a diagnosis of multiple primary neoplasms (bilateral or multiple cancers). Additionally, relevant tumor characteristics were considered, including the HER2-negative subtype in BC, due to its known association with *BRCA2* pathogenic variants.

The guidelines provided in the Declaration of Helsinki were followed to ensure the welfare and rights of the study participants. All patients were informed about the objectives and procedures of the study, and their written informed consent was obtained before sample collection. The study protocol was approved by the ethics committee under registration number R-2022-1305-114 at the Centro de Investigación Biomédica de Occidente, Instituto Mexicano del Seguro Social (CLIES #1305), ensuring compliance with all relevant ethical and legal regulations.

### 2.2. Identification of Variants in the BRCA2 Gene

#### 2.2.1. Genomic DNA Extraction

Genomic DNA was extracted from peripheral blood samples using the AmoyDx Blood/Bone Marrow Spin Column kit (AmoyDX, Singapore), strictly following the manufacturer’s protocol. The quality and concentration of the extracted DNA were assessed using spectrophotometry (Qubit 4, ThermoFisher Scientific, Waltham, MA, USA) and agarose gel electrophoresis to ensure its integrity and purity before proceeding with downstream analyses.

#### 2.2.2. BRCA2 Sequencing

The complete coding regions of the *BRCA2* gene were targeted for analysis. Selective capture and amplification of these regions were performed using amplicon enrichment technology with the commercial AmpliSeq for Illumina BRCA Panel. This panel is specifically designed to comprehensively cover the coding exons and splice site boundaries of *BRCA1* and *BRCA2*, enabling accurate detection of single nucleotide variants, insertions, deletions, and splice-affecting alterations. DNA libraries were prepared following the standard AmpliSeq protocol, which includes amplification, purification, and quantification steps. Subsequently, libraries were sequenced on the Illumina MiSeq next-generation sequencing platform (Illumina, Inc., San Diego, CA, USA) using transcripts of the variants *BRCA2* (NM_000059.3) with a compatible sequencing kit that generated high-quality, appropriately long reads for detailed analysis.

#### 2.2.3. Bioinformatic Analysis and Variant Annotation

Sequence reads were processed and variants identified using the ANDAS-Amoy platform, a specialized tool that performs sequence alignment, variant calling, and functional annotation. Variants were filtered based on quality metrics and a minor allele frequency (MAF) of ≤0.01, as per the gnomAD and ClinVar databases. Potentially damaging variants were prioritized based on their predicted pathogenicity by in silico tools integrated into the platform. Additional annotations included genomic position, predicted protein effect, and allele frequency in public databases. The analysis focused on coding variants; no splice site variants meeting these criteria were identified in the cohort.

#### 2.2.4. Variant Classification and Evaluation

Clinical interpretation of detected variants was conducted according to the guidelines established by the ACMG/AMP consortium [22]. This framework categorizes variants as pathogenic, likely pathogenic, variants of uncertain significance, likely benign, or benign.

### 2.3. Computational Analysis of Variants

#### 2.3.1. Structural Identification of Functional Domains Using UniProt

To characterize the structural localization of a set of variants in the *BRCA2* gene, the UniProt database [23] (https://www.uniprot.org, accessed on 20 June 2025) was utilized through its official web platform. The entry corresponding to the identifier P51587, representing the BRCA2 protein encoded by the NM_000059.4 transcript, which was selected as part of the MANE Select set, was consulted. From this entry, functional domains and structural regions of the protein were identified, along with their corresponding annotations.

UniProt [23] is a comprehensive database that provides detailed protein sequence and functional information, including annotations on protein function, structure, and involvement in diseases. It is a key resource for researchers in the fields of genomics and bioinformatics.

For each analyzed variant, the corresponding position of the amino acid change in the protein sequence was located and manually compared with the annotated region ranges. This allowed for determining whether the variant resided within a functional domain, an unstructured region, or in areas with no known annotation. This approach enabled the classification of each variant based on its relative location to critical domains of the BRCA2 protein, providing an initial assessment of its potential functional relevance.

#### 2.3.2. Functional Analysis and Impact Prediction Using Ensembl VEP

Subsequently, a more detailed functional annotation was performed using the Variant Effect Predictor (VEP) tool, version 115, from Ensembl [24] (https://useast.ensembl.org/info/docs/tools/vep/index.html, accessed on 26 June 2025), which is available online via its web interface. The reference transcript NM_000059.4, corresponding to the *BRCA2* gene and recognized as the MANE Select transcript, was used. Variants were analyzed in rsID format when available, and in HGVS cDNA format when the polymorphism was not registered in databases.

VEP version 115 [24] is a tool used to predict the functional effects of genetic variants. It provides detailed annotations for variants, including their impact on protein-coding regions, regulatory regions, and noncoding sequences. VEP integrates data from multiple sources, including gene models, regulatory features, and known pathogenic variants, to help researchers assess the potential consequences of genetic variations on gene function and disease.

For each entered variant, VEP version 115 [24] provided information on the type of molecular consequence (such as missense_variant, frameshift_variant, or stop_gained), the estimated functional impact (classified as high, moderate, or low), codon changes, and alignment with the selected transcript. Additionally, computational predictions generated by the SIFT [25] and PolyPhen version 2 (PolyPhen-2) [26] algorithms, which estimate whether a nonsynonymous variant could alter protein function or structure, were included. SIFT and PolyPhen-2 predictions are only available for missense variants.

SIFT [25] classifies variants as deleterious or tolerated based on the evolutionary conservation of the affected residues. At the same time, PolyPhen-2 [26] categorizes them as probably damaging, possibly damaging, or benign, based on structural and functional criteria. Both tools also provide a quantitative score between 0 and 1, indicating the confidence in the prediction. The results were recorded in a comparative table for each variant, integrating information about their localization in functional domains with their functional predictions, to facilitate biological and clinical interpretation.

### 2.4. Structural Impact Prediction and Local Visualization of Variants

#### 2.4.1. Structural Impact Prediction

To explore the possible structural consequences of the identified missense variants in *BRCA2*, the HOPE tool (Have (y)Our Protein Explained) version 1.1.1 [27] was used (https://www3.cmbi.umcn.nl/hope/, accessed on 19 April 2025). This resource enables automated analysis based on the physicochemical properties of amino acids, evolutionary conservation information, and annotations from UniProt. Due to the lack of a resolved three-dimensional structure for the complete BRCA2 protein, the analysis was performed based on computational predictions and sequence alignments, using the UniProt identifier P51587 as a reference for the protein encoded by the NM_000059.4 transcript (MANE Select).

For each missense variant, HOPE [27] provided a comparison between the wild-type and mutant amino acids in terms of size, charge, and hydrophobicity, as well as indicating whether the affected residue was evolutionarily conserved. Additionally, when possible, annotations on proximity to functional regions or previously reported variants in databases were provided. The tool generated an automated diagnosis of the potential structural or functional disruption caused by the change, based on the biochemical differences between the residues.

#### 2.4.2. Fragment Modeling

To illustrate the precise localization of selected missense variants in the BRCA2 protein, three-dimensional models of local fragments of approximately 20 amino acids centered on the position of each amino acid change were generated. This modeling was performed exclusively for missense variants, regardless of their clinical classification, in order to visualize their immediate structural environment. The corresponding sequences were obtained from the UniProt entry ID P51587, corresponding to the canonical isoform of BRCA2. The fragments were submitted to the AlphaFold Server, using the AlphaFold version 3 structure prediction model (AlphaFold3) [28] (https://alphafoldserver.com/, accessed on 30 June 2025). Models were generated for both the wild-type and mutated sequences, with the residue corresponding to each variant manually modified. The resulting models were visualized using UCSF ChimeraX version 1.9 [29] (https://www.cgl.ucsf.edu/chimerax/, accessed on 30 June 2025), where mutated residues were highlighted using various representation styles, such as “stick” and “sphere,” along with text labels. This structural analysis was performed solely for illustrative purposes to show the immediate environment of each variant, without making functional or protein stability inferences; therefore, confidence metrics such as pLDDT values were not considered in this work.

AlphaFold [28] is an artificial intelligence–based computational tool developed by DeepMind that enables the high-accuracy prediction of protein three-dimensional structures from their amino acid sequences. It utilizes deep learning models trained on known structures to infer the most probable conformation of the polypeptide chain. Its use has become widespread in structural bioinformatics studies due to its ability to generate reliable models even in the absence of experimental data. ChimeraX [29], on the other hand, is a molecular visualization software developed by the University of California, San Francisco (UCSF), designed for the analysis, editing, and graphical representation of biomolecular structures. Its intuitive interface and capacity to work with both predicted models and experimental structures make it a key tool for exploring molecular interactions, locating variants, and generating high-quality figures for scientific publications.

It is important to note that the Structural Impact Prediction and Local Visualization of Variants analysis was applied only to missense variants, as stop-gained variants are assumed to disrupt protein formation.

## 3. Results

### 3.1. Sociodemographic and Clinicopathological Characteristics of the Study Patients

A total of 140 patients were included in this study, comprising 116 women with BC, 19 with ovarian cancer (OC), and 5 with combined BC and OC diagnoses (Table 1 and Table 2). Regarding sociodemographic characteristics (Table 1), the mean ages were 46.9 ± 13.3 years (range, 20–79) for BC patients, 56.6 ± 11.1 years (range, 35–77) for OC patients, and 49.0 ± 10.7 years (range, 33–61) for the combined group. A statistically significant age difference was observed between the BC and OC groups (*p* = 0.028), with patients diagnosed with OC being, on average, older. The combined group was excluded from the comparative analysis due to its small sample size. The age at menarche averaged 12.2 ± 1.56 years (range, 8–16) in BC, 11.8 ± 0.89 years (range, 10–13) in OC, and 12.4 ± 0.54 years (range, 12–13) in the combined group. Regarding BMI, normal weight was predominant in BC (76%) and OC (58%), while all patients in the combined group were obese (100%). Alcohol, tobacco consumption, and hormone therapy were reported as negative in most groups. Pre-menopause status (53%) was more frequent in the BC, in contrast to the OC group (84%) and the combined group (60%). Breastfeeding and the family history of cancer in first or second degree relatives with breast, ovarian, prostate, or pancreatic cancer were reported as positive in most groups. The autodetection was characteristic of the BC, and sonogram, ultrasonogram in the OC, and the combined group.

Clinicopathological characteristics (Table 2) revealed that the study groups were predominantly diagnosed between 1 and 4 years. The tumors were predominantly unilateral. The most frequent clinical stages in BC were II and III. Histologically, invasive ductal carcinoma was the predominant type in BC (94%), whereas high-grade serous carcinoma was universal in OC and the combined group (100%). Regarding molecular subtypes, 44% of breast tumors were triple negative; all ovarian and combined cases were high-grade serous, with combined cases showing serous with Luminal A and serous with triple negative subtypes. In the three study groups, most patients had a Ki-67 proliferation index of 20% or greater. Lymph node involvement was observed in 40% of BC patients and 21% of OC patients. Treatment response was complete in 63% of BC cases, partial in 42% of OC cases, and complete in 80% of the combined group.

### 3.2. Frequency and Distribution of BRCA2 Variants in Patient Cohorts

All 140 patients included in this study underwent successful *BRCA2* gene sequencing. Variants were identified in 18 patients (12.86%), including 14 women with BC (12%) and four patients with OC (21.05%). The variants detected in the studied patients are detailed in Table 3.

### 3.3. Comparison of Patients Carrying and Not Carrying BRCA2 Variants

When the clinical characteristics of the study groups were compared, stratified by *BRCA2*-positive and BRCA2-negative status for each cancer type, the cohort included 102 *BRCA2*-negative and 14 *BRCA2*-positive BC cases, as well as 15 *BRCA2*-negative and 4 *BRCA2*-positive OC cases (Table S1). In BC, statistically significant differences were observed for overweight status (29% vs. 6%, *p* = 0.019), family history of first-degree relatives with breast/ovarian/pancreatic cancer (100% vs. 57%, *p* = 0.004), and chemotherapy response (complete response 36% vs. 69%, *p* = 0.032). In OC, significant associations were found for abortion history (0% vs. 80%, *p* = 0.018) and laterality (bilateral cases 75% vs. 13%, *p* = 0.037) (Table 4).

It is worth noting that the variants rs80359380, rs587780646, rs397507422, and rs11571658 have been previously reported in the Mexican population among patients with BC. All of these previously reported variants showed no statistically significant differences in frequency compared to the population included in this study (*p* > 0.05), except for rs11571658. In contrast, the variants rs587782313, c.3481_3482dup, and rs80359479 have been previously reported in populations from Portugal and Brazil, respectively. Significant differences in allele frequencies were observed when compared to our study population (*p* < 0.05). Finally, the variants rs398122715, rs1329182873, c.9812T>C, rs775030825, rs1064795067, and rs80359219 have not been reported in other cohorts. However, we observed significant differences in their frequencies compared to those reported in the gnomAD and dbSNP databases (Table 5).

### 3.4. Computational Analysis

#### 3.4.1. Structural Localization of Variants in Functional Domains of BRCA2

**Figure 1:**
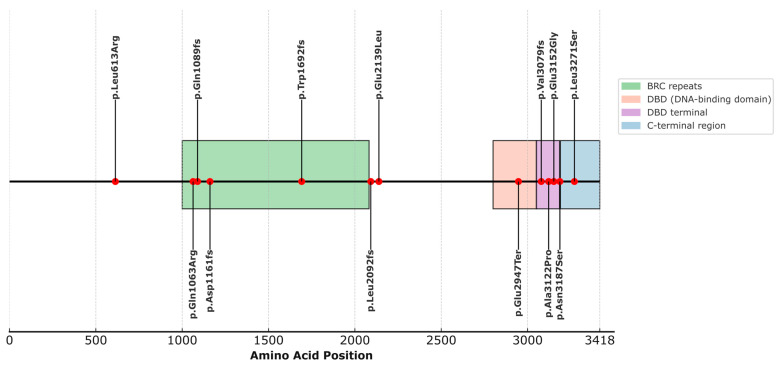
Distribution of BRCA2 variants across annotated protein domains.

When analyzing the structural localization of the selected variants in the BRCA2 protein, we observed that most of them fall within well-defined functional domains, as indicated by the available annotation in UniProt (ID: P51587) (Figure 1, Table S2). Specifically, four variants are located within the domain known as BRC repeats (1003–2082), which play essential roles in RAD51 binding for the formation of nucleofilaments during homologous recombination repair. These include p.Gln1089fs (rs80359380), p.Gln1063Arg (rs775030825), p.Asp1161fs (c.3481_3482dup), and p.Trp1692fs (rs80359479). The variant p.Glu2947Ter (rs398122715) is located within the DNA-binding domain (DBD) (2804–3054), a crucial domain for the interaction of BRCA2 with single-stranded and double-stranded DNA, as well as facilitating binding to the regulatory protein DSS1. Variants p.Ala3122Pro (rs587782313), p.Val3079Phefs (rs397507422), and p.Glu3152Gly (c.9455A>G) are located in the terminal portion of the DBD, within a structurally recognized region known as the terminal DBD (3052–3185), with essential functions in the stability of the repair complex. The variant p.Asn3187Ser (rs1329182873) is located in the C-terminal region/DBD boundary (3190–3418) and is involved in phosphorylation processes, nuclear localization, and interaction with RAD51. On the other hand, variants p.Leu613Arg (rs587780646), p.Leu2171Ser (c.9812T>C), p.Leu2092fs (rs11571658), and p.Glu2139Leu (c.6415_6416delinsAT) are not localized within any currently described functional domain (Figure 1).

#### 3.4.2. Functional Analysis via VEP Annotation

The analysis of selected *BRCA2* variants using the VEP revealed a range of molecular consequences, computational predictions, and relevant clinical annotations, summarized in Table 6. A large proportion of the variants were classified as missense variants, involving single amino acid substitutions. Most of these missense changes were predicted to be deleterious by SIFT, with scores close to zero, indicating a likely detrimental effect on protein function. PolyPhen predictions showed greater variability, with some alleles classified as benign, while others were predicted to be possibly damaging or probably damaging, reflecting context-dependent structural effects. Similarly, variants like rs80359380, rs80359479, and rs11571658 were identified as frameshift variants, disrupting the reading frame and associated with clinical annotations ranging from pathogenic to uncertain significance. The clinical significance annotations from ClinVar were heterogeneous, with many variants annotated as of uncertain significance or displaying conflicting interpretations of pathogenicity. This highlights the ongoing challenges in the clinical interpretation of *BRCA2* variants. For example, rs775030825 and rs587780646 share missense consequences but differ in their computational predictions and clinical classifications, ranging from uncertain significance to likely benign. Variants c.9812T>C (p.Leu3271Ser) and c.3481_3482dup (p.Asp1161fs) are not annotated in VEP; however, they can be manually classified as missense and frameshift variants, respectively.

**Figure 2:**
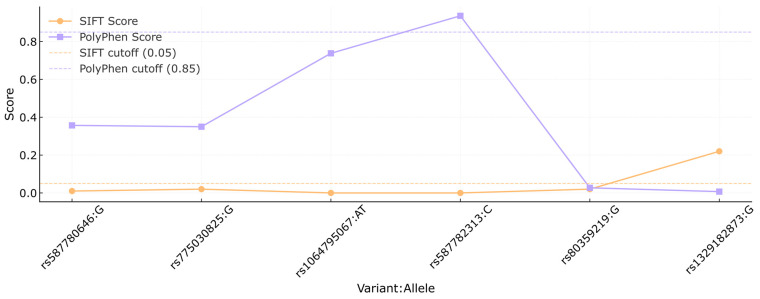
Comparison of SIFT and PolyPhen scores of the *BRCA2* variants. Each dot represents the score assigned to a specific variant (shown as rsID:allele) by the SIFT (orange) and PolyPhen (purple) algorithms. Dashed lines indicate the cutoff thresholds for predicting deleterious effects: 0.05 for SIFT and 0.85 for PolyPhen. This analysis was conducted to assess the potential functional impact of missense variants based on in silico predictions.

In Figure 2, the SIFT and PolyPhen scores are compared for each variant. The orange line represents the SIFT scores, while the PolyPhen scores are shown in purple. Cutoff thresholds for SIFT (0.05) and PolyPhen (0.85) are marked by the orange and purple dashed lines, respectively, to indicate the functional impact ranges for each score. Variants rs80359380, rs80359479, rs11571658, rs398122715, rs397507422, c.9812T>C, and c.3481_3482dup had no reports available in the previously mentioned platforms.

#### 3.4.3. Structural Consequences of the Variants

The seven missense variants in the *BRCA2* gene were analyzed using the HOPE tool (Table 7), which focuses on predicting structural consequences based on amino acid properties. Four variants (rs587780646, rs1329182873, rs587782313, and c.9812T>C) affect highly conserved residues, with predictions indicating alterations in charge, size, and/or hydrophobicity, potentially compromising protein architecture or its molecular interactions. In contrast, the variant rs775030825 (p.Gln1063Arg) does not affect a conserved residue, and its substitution is observed in other species, making HOPE less likely to predict a detrimental effect.

**Figure 3:**
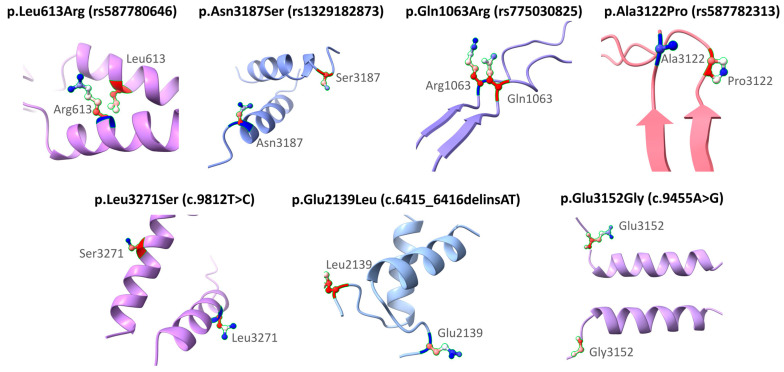
Structural representation of missense variants in the *BRCA2* gene.

Figure 3 shows the structural modeling of seven missense variants in the *BRCA2* gene using AlphaFold. The original and substituted residues are displayed within their local three-dimensional structural context. The variants included are p.Leu613Arg (rs587780646), p.Asn3187Ser (rs1329182873), p.Gln1063Arg (rs775030825), p.Ala3122Pro (rs587782313), p.Leu3271Ser (c.9812T>C), p.Glu2139Leu (c.6415_6416delinsAT), and p.Glu3152Gly (c.9455A>G). Each substitution is mapped onto the corresponding secondary structure model to visualize its position and the nature of the amino acid change. This modeling was performed solely for illustrative purposes and does not aim to predict functional or clinical consequences. The clinical interpretations of these variants are based on ACMG classification and other computational analyses described in previous sections. Frameshift variants (rs80359380, rs80359479, rs11571658, and rs397507422) were not modeled because they are predicted by VEP to generate truncated proteins (Table 6).

### 3.5. Proposed Prioritization of BRCA2 Variants

The *BRCA2* variants identified in this study were organized according to their domain localization, computational predictions, and reported clinical classification. Based on these parameters, we propose a prioritization that may help to guide subsequent functional or clinical investigations. Table 8 summarizes the integration of these data for all variants analyzed.

## 4. Discussion

In Mexico, as in other regions of the world, BC and OC represent a significant public health issue due to their increasing incidence and mortality rates. BC ranks first, and OC second, in terms of incidence and mortality among gynecological cancers in the country [1,37]. The frequency of BC is higher in women around 50 years of age, an age considered productive, which is also reflected in a higher mortality rate within this age group [38]. On the other hand, the average age at diagnosis for OC is estimated to be around 63 years [39]. The prevalence and impact of both types of cancer in Mexico and worldwide depend on multiple factors, including genetic predisposition, lifestyle, and access to healthcare services [5,39,40].

Regarding the sociodemographic characteristics observed in the groups analyzed in this study (Table 1), the average age of patients was similar to that reported in the literature [38,39]: 49.0 ± 10.7 years in the group with both BC and OC, 46.9 ± 13.3 years in the BC group, and 56.6 ± 11.1 years in the OC group. Menopausal status was more frequent in the OC and combined groups, while premenopausal status predominated in the BC group. Clinically (Table 2), BC was unilateral in most cases, recently diagnosed (1 to 4 years), of invasive ductal type, and notably presented a triple-negative profile in 44% of cases. This overrepresentation of TNBC, consistent with the study’s inclusion criteria emphasizing HER2-negative subtypes, should be acknowledged as a potential source of selection bias in the clinical profile of our BC cohort. OC cases were predominantly high-grade serous, also characterized by elevated Ki-67 levels, and were diagnosed at advanced stages; however, the small number of OC cases in our series limits the generalizability of these clinicopathological observations. Likewise, the subgroup of patients diagnosed with both BC and OC was very small, and the clinical patterns observed in this group should be interpreted as anecdotal.

Advances in the understanding of BC and OC have led to improvements in health policies, resulting in better quality of care and earlier detection for patients with these conditions. However, there is still a noticeable trend of self-detection of BC, often at advanced stages.

Since BC and OC are multifactorial diseases, various risk factors may influence their development [4,39,40]. Nevertheless, genetic contribution has been recognized as a key factor in the susceptibility to these tumors. It is estimated that 5 to 10% of BC and OC cases are hereditary, and that germline variants in genes such as *BRCA1* and *BRCA2* account for a significant proportion of these cases [41,42]. Specifically, variants in these genes increase the risk of developing BC by more than 50% and OC by up to 40% [43,44].

The crucial role of BRCA proteins in the cell cycle and DNA repair through homologous recombination highlights the importance of studying these genes [9,45]. Moreover, variants in these genes are considered dominant, which means they can increase cancer risk even in the heterozygous state. These variants have also been associated with other cancer types beyond BC and OC, including prostate, pancreatic, and other malignancies [44,46,47,48].

The detection of variants in the *BRCA* genes is a well-established and widely available method for families at risk of breast, ovarian, prostate, and pancreatic cancers in developed countries [49]. Thousands of variants associated with these cancer types have been described. Most of these variants lead to premature stop codons, caused by deletions, insertions, or base substitutions, resulting in the early termination of the protein. These so-called deleterious variants are often located in critical sequence regions that contain recognition sites for functional motifs of the protein [50,51]. However, the functional effect of many of these variants remains unknown. The reported frequency of *BRCA2* variant carriers in the general population is approximately 1 in 800 individuals [52], with a higher prevalence observed in the Ashkenazi Jewish population [53].

The management of patients carrying pathogenic or likely pathogenic *BRCA2* is individualized and may include increased surveillance, chemoprevention with tamoxifen, bilateral prophylactic oophorectomy, and/or bilateral prophylactic mastectomy [54,55]. This approach encompasses the etiology, epidemiology, pathophysiology, screening, evaluation, and management of *BRCA2* variants, and highlights the importance of an interprofessional team in educating patients about cancer risk and appropriate management strategies.

In our analysis (Table 3), thirteen *BRCA2* variants were identified in Mexican women with BC (n = 14) and OC (n = 4), resulting in a carrier frequency of 12.86%. The frequency was higher in the OC group (27.8%) compared to the BC group (12%). Although this difference was not statistically significant, it is consistent with studies showing that some *BRCA2* variants are more frequently associated with OC [56].

When we compared the clinical and pathological characteristics of carrier patients with non-carrier patients, we observed that, in BC, *BRCA2* carriers were more frequently overweight, had a more marked family history of breast, ovarian, or pancreatic cancer, and showed a lower complete response rate to chemotherapy, suggesting both a hereditary influence and possible differences in treatment sensitivity. In OC, carriers more frequently presented with bilateral cases, consistent with hereditary patterns, while the absence of a history of miscarriages should be interpreted with caution due to the small sample size. Overall, these findings highlight the clinical relevance of *BRCA2* variant status in patient presentation and outcomes.

Among the identified variants, frameshift, missense, and one truncating variant were reported. Several of these, such as rs80359380, rs80359479, rs11571658, rs397507422, rs878853569, rs80359479, and rs398122715, have been previously classified as pathogenic. The variant rs587782313 was classified as likely pathogenic. Additionally, six VUS were identified, reflecting the challenges of clinical interpretation, particularly in underrepresented populations such as the Mexican population. These VUS included rs775030825, rs1064795067, rs80359219, rs1329182873, c.9812T>C, and rs587780646. VUS are typically characterized by their low population frequency, lack of direct functional evidence, location outside well-defined functional domains, and conflicting annotations in clinical databases such as ClinVar [57,58]. This highlights the need for complementary analyses to clarify their functional impact.

The in silico analysis allowed us to characterize these variants from both structural and functional perspectives (Table 6 and Table 7). For example, rs587782313, located in the terminal region of the DNA-binding domain (DBD), showed consistent predictions of deleterious effect in SIFT (0.0), probably damaging in PolyPhen (0.936), and structural alterations according to HOPE. Its position in a critical region for DSS1 interaction and DNA binding further supports its potential functional impact. Similarly, rs1064795067, although outside a canonical domain, showed an unfavorable profile across all tools, suggesting disruption of salt bridges due to the loss of negative charge. Other variants, such as rs587780646, located outside known domains, presented mixed predictions across SIFT, PolyPhen, and HOPE. Despite not falling within classic functional regions, it has been proposed that the intermediate regions of BRCA2 may have regulatory roles that are not yet fully characterized [9]. These observations suggest that current structural annotation may still be incomplete, particularly in large and complex proteins such as BRCA2.

A critical finding was that four of the six VUS identified in our research (rs775030825, rs80359219, rs1329182873, and c.9812T>C) were located within functionally relevant regions (such as the BRC repeats and the DBD), highlighting the need to re-evaluate these classifications based on functional and structural evidence. This pattern is also observed in other Latin American populations, where variants classified as rare or uncertain in European databases appear at higher frequencies, suggesting a distinct ethnic and genomic background [16,17,32,36]. For example, variants such as rs80359380 and rs11571658 were significantly more frequent in our cohort (2.1% and 1.4%, respectively) compared to their global frequencies reported in dbSNP (0.000012% and 0.000753%, respectively).

We applied a stepwise rubric that integrates variant type, domain localization, concordant in silico predictions and ClinVar annotation. This approach organizes the thirteen *BRCA2* variants into high, moderate, and low priority categories to guide follow-up.

High priority included truncating changes p.Gln1089fs (rs80359380), p.Trp1692fs (rs80359479), p.Asp1161fs (rs878853569), p.Glu2947Ter (rs398122715), p.Val3079fs (rs397507422), and p.Leu2092fs (rs11571658). All are expected to abolish essential motifs, including BRC repeats or the DNA-binding domain. ClinVar reports these variants as pathogenic, frequently with expert panel review, which is entirely consistent with their high priority in our rubric. The missense p.Ala3122Pro (rs587782313) also reached high priority due to its location in the terminal DNA-binding domain and concordant deleterious predictions. ClinVar is conflicting for this variant (uncertain significance versus likely pathogenic). Our designation, therefore, diverges from current clinical consensus but is supported by the convergence of domain and computational evidence.

Moderate priority comprised p.Glu3152Gly (rs80359219), p.Glu2139Leu (rs1064795067), p.Leu613Arg (rs587780646), and p.Gln1063Arg (rs775030825). For p.Glu3152Gly, the location of the terminal DNA-binding domain is relevant, although PolyPhen is classified as benign, and ClinVar lists uncertain significance. This aligns with a moderate assignment. For p.Glu2139Leu and p.Leu613Arg, both lie outside annotated domains but show deleterious SIFT and HOPE-suggested perturbations; ClinVar provides conflicting or likely benign signals, which support an intermediate position rather than a high or low one. For p.Gln1063Arg, ClinVar is conflicting, and HOPE suggests a limited impact; however, the location within BRC repeats and a deleterious SIFT score justify a moderate level under this rubric.

Low priority included p.Asn3187Ser (rs1329182873) and p.Leu3271Ser (no rsID). These variants show predominantly benign or non-concordant predictions and lack strong domain anchoring. ClinVar lists p.Asn3187Ser as uncertain significance with no conflicts, which is consistent with a low assignment. p.Leu3271Ser lacks VEP annotation and has no consolidated ClinVar entry in our dataset; the low level reflects limited current evidence and indicates the need for data accrual rather than immediate functional testing. Nevertheless, the absence of experimental validation or orthogonal evidence (e.g., functional assays, family segregation, or tumor sequencing) limits the robustness of these prioritization levels. Integrating such data in future studies will be essential to confirm or refine the functional effect of the variants.

It is worth noting that several of the variants detected in this study have been previously reported in other populations, which supports their potential involvement in hereditary cancer. For instance, rs587782313 has been described in patients with OC in Portugal [30], while rs80359380 has been reported in both Spanish [31] and Mexican cohorts [32], and rs80359479 and c.3481_3482dup in Brazilian patients [33,34]. Likewise, variants such as rs587780646, rs397507422, and rs11571658 have been previously reported in studies conducted in the Mexican population [16,17,36], suggesting that these variants may recur in specific population subgroups.

In our analysis, most of these previously reported variants did not show significant differences in frequency compared to the present cohort, with the exception of rs80359380 and rs11571658, which were significantly different when contrasted with Spanish and Canadian studies, respectively. Conversely, variants such as rs587782313, c.3481_3482dup, and rs80359479 did not differ from other cohorts but showed significant differences when compared with gnomAD data. In addition, several variants not previously reported in other cohorts (rs398122715, rs1329182873, c.9812T>C, rs775030825, rs1064795067, and rs80359219) also displayed significant differences relative to gnomAD (Table 5). These comparisons should be interpreted with caution, as study design, sample size, and population ancestry may influence observed frequencies; however, they provide valuable context to highlight potential population-specific effects.

Regarding the six of the thirteen variants identified in this study (rs398122715, rs1329182873, c.9812T>C, rs775030825, rs1064795067, and rs80359219) that have not been previously reported in the Mexican population or other described cohorts, this study represents the first time these variants have been documented in this population, underscoring the need for ongoing evaluation of their potential clinical significance. Their low frequency in global databases such as gnomAD, along with their absence in prior regional and international studies, supports the hypothesis that these may represent rare but potentially significant events in the Mexican genomic context.

This observation becomes even more relevant considering that, among these previously unreported variants, all except rs398122715 are currently classified as VUS. These findings underscore the importance of conducting genetic studies in underrepresented populations, such as those in Latin America, to broaden our understanding of genetic variability in susceptibility genes like BRCA2. Furthermore, they highlight the need to update international databases with variants identified in diverse ethnic contexts, as this could significantly improve the clinical interpretation of variants currently classified as VUS.

When compared with independent studies in the Mexican population, our results for *BRCA2* in Jalisco complement and expand the national mutational landscape. The systematic review by Alday-Montañez et al. (2024) [59] aggregated 9026 Mexican genotypes and documented 657 pathogenic variants, reporting that while BRCA1 accounts for a larger proportion of hereditary breast and ovarian cancer cases, *BRCA2* harbors recurrent and regionally enriched variants, particularly in exon 11. Similarly, Catalán et al. (2019) [60] described 12 distinct *BRCA2* pathogenic variants in Mexican families, several of which were not previously reported in Latin America, underscoring the ongoing discovery of *BRCA2* alleles in the country. In ovarian cancer, Gallardo-Rincón et al. (2020) [61] identified germline *BRCA2* variants in approximately one-third of mutation-positive patients, highlighting clinically relevant differences in outcome compared to BRCA1 carriers. Our study contributes to this national context by characterizing *BRCA2* variants in a regional (Jalisco) cohort, including six not previously reported in Mexican or other populations. These findings expand the catalog of *BRCA2* diversity and provide population-specific information that can aid in clinical interpretation. Nevertheless, given the consistent evidence that BRCA1 represents a larger share of the hereditary burden in Mexico, future research in Jalisco should integrate BRCA1, particularly screening for the exon 9–12 founder deletion.

Taken together, our study, which integrated clinical, population-based, and computational data, provides a more comprehensive view of the potential functional and clinical impact of *BRCA2* variants in Mexican patients with breast and ovarian cancer. This strategy enabled us to address not only the frequency and distribution of the variants but also their possible biological effects by incorporating structural predictions, localization within functional domains, and associated clinical evidence. This approach is particularly relevant for VUS, whose interpretation remains a persistent challenge in oncological and genetic practice. The multidimensional contextual analysis of these variants offers a stronger foundation for generating hypotheses about their potential pathogenicity and suggests clear directions for their future reclassification.

Moreover, our findings underscore the need to characterize genetic variants in underrepresented populations, such as the Mexican population, where ancestral backgrounds, local evolutionary dynamics, and admixture profiles may give rise to unique variants or result in different allele frequencies compared to those reported in international databases. The identification of several VUS in functionally relevant regions of the *BRCA2* gene, combined with their limited representation in global cohorts, reveals a significant gap in current knowledge and constrains clinical interpretation in these populations. This supports the need for comprehensive functional studies to assess the impact of these variants on protein structure, stability, and function, as well as on DNA repair mechanisms mediated by homologous recombination.

Likewise, the importance of conducting validations in larger and genetically diverse cohorts is emphasized, as such efforts would allow for more reliable associations between specific variants and phenotypic traits such as histological type, age at diagnosis, or family history of cancer. These studies would help mitigate the interpretation bias arising from the overreliance on data from European populations and improve the predictive power of genetic panels in Latin American settings.

Finally, we propose working toward the inclusion of these variants in clinical genetic screening panels tailored to the Mexican context, in both public and private healthcare systems. This would enhance early diagnosis, improve the stratification of hereditary cancer risk, guide preventive or surgical management decisions, and expand therapeutic options based on individual molecular profiles. Integrating these findings into clinical practice would contribute to the development of a more equitable, inclusive, and ethnically contextualized precision medicine, aligned with the specific needs of the Mexican population and other Latin American communities.

## 5. Conclusions

*BRCA2* variants were identified in 12.86% of patients, with a higher prevalence in ovarian cancer. Several affected key functional domains and showed functional and structural profiles consistent with pathogenic effects. Notably, six had not been previously reported in other populations, highlighting the distinct genomic background of the Mexican population. These findings underscore the urgent need to develop population-specific genetic screening panels that incorporate locally relevant variants to enhance diagnostic accuracy and inform clinical decision-making in hereditary cancer care.

## Figures and Tables

**Table 1:** Sociodemographic characteristics of the cohort of patients.

Variable		Breast Cancer		Ovary Cancer		Breast and Ovary Cancer	
Number of Patients		116		19		5	
Age (years) *							
	media ± SD **	46.9 ± 13.3		56.6 ± 11.1		49.0± 10.7	
	Range	20–79		35–77		33–61	
Age at Menarche (years)							
	media ± SD *	12.2 ± 1.56		11.8 ± 0.89		12.4 ± 0.54	
	Range	8–16		10–13		12–13	
BMI (Body Mass Index) kg/m^2^							
	Normal 18.5 ± 25 (*n*, %)	88	(76)	11	(58)	----	----
	Overweight 25.1 ± 30 (*n*, %)	20	(17)	5	(26)	----	----
	Obese > 31.9 (*n*. %)	8	(7)	3	(16)	5	(100)
Alcohol Consumption							
	Yes (*n*. %)	19	(16)	5	(26)	----	----
	No (*n*. %)	97	(84)	14	(74)	5	(100)
Tobacco Consumption							
	Yes (*n*. %)	17	(15)	3	(16)	----	----
	No (*n*. %)	99	(85)	16	(84)	5	(100)
Hormonal Consumption							
	Yes (*n*. %)	41	(35)	----	----	1	(20)
	No (*n*. %)	75	(65)	19	(100)	4	(80)
Menopause Status							
	Pre-menopause (*n*. %)	62	(53)	3	(16)	2	(40)
	menopause (*n*. %)	54	(47)	16	(84)	3	(60)
Abortion							
	Yes (*n*. %)	18	(16)	5	(26)	----	----
	No (*n*. %)	98	(84)	14	(74)	5	(100)
Breastfeeding							
	Yes (*n*. %)	79	(69)	14	(74)	3	(60)
	No (*n*. %)	36	(31)	5	(26)	2	(40)
Family History of Cancer							
	First or second degree relative with breast, ovarian, or pancreatic cancer (*n*. %)	74	(64)	13	(89)	3	(60)
	First or second degree relative with other cancer type (*n*. %)	16	(14)	3	(11)	1	(20)
	No (*n*. %)	26	(22)	3	(11)	1	(20)
Cancer Detection							
	Autodetection (*n*. %)	107	(92)	----	----	----	----
	sonogram, ultrasonogram (*n*. %)	9	(8)	19	(100)	5	(100)

* Age BC vs. OC *p* = 0.003, ** Standard Deviation (SD). Obesity OC vs. OC/BC groups were *p* = 0.0000.

**Table 2:** Clinicopathological characteristics of the cancer shown by the patients.

Variable	Breast Cancer (*n* = 116)		Ovary Cancer (*n* = 19)		Breast and Ovary Cancer (*n* = 5)	
	*n*	(%)	*n*	(%)	*n*	(%)
Time since diagnosis (years)						
1–4	84	(72)	13	(68)	2	(40)
5–9	14	(12)	6	(32)	1	(20)
10 and more	18	(16)	--	--	2	(40)
Laterality						
Unilateral	104	(90)	17	(89)	1	(100)
Bilateral	12	(10)	2	(11)	--	--
Clinical Stage						
In situ	3	(3)	--	--	--	--
I	19	(16)	2	(10)	--	--
II	36	(31)	6	(32)	3	(60)
III	43	(37)	7	(37)	2	(40)
IV	15	(13)	4	(21)	--	--
Histology						
Ductal	109	(94)	--	--	--	--
Lobulillar	6	(5)	--	--	--	--
Mixto	1	(1)	--	--	--	--
High-grade serous	--	--	19	(100)	--	--
Ductal/High-grade serous	--	--	--	--	5	(100)
Molecular subtype						
Luminal A	23	(20)	--	--	--	--
Luminal B	30	(26)	--	--	--	--
Triple negative	51	(44)	--	--	--	--
Luminal A/B	4	(3)				
Triple negative/Luminal A	8	(7)	--	--	--	--
High-grade serous	--	--	19	(100)	--	--
High-grade serous/triple negative	--	--	--	--	3	(60)
High-grade serous/Luminal A	--	--	--	--	2	(40)
KI-67						
<20%	21	(18)	--	--	1	(20)
≥20%	95	(82)	19	(100)	4	(80)
Lymph Node						
positive	46	(40)	4	(21)	--	--
negative	70	(60)	15	(79)	4	(100)
Chemotherapy response						
Complete	72	(63)	8	(42)	4	(80)
Partial	34	(29)	8	(42)	--	--
No response	4	(3)	1	(5)	--	--
Recurrency	6	(5)	2	(11)	1	(20)

**Table 3:** Genetic *BRCA2* variants detected among BC and OC cases.

HGVS Nomenclature (cDNA Level)	HGVS Nomenclature (Protein)	(rsID)	Frequency *n* (%) *	Type of Cancer	Clinical Classification	Clinvar Classification (Review Status ****)
NM_000059.3(*BRCA2*):c.3188A>G	(p.Gln1063Arg)	rs775030825	1 (0.7)	BC	VUS **	Conflicting classifications of pathogenicity (Guidelines based)
NM_000059.4(*BRCA2*):c.3264dup	(p.Gln1089fs)	rs80359380	3 (2.1)	BC	Pathogenic	Pathogenic (Reviewed by expert panel, guidelines-based)
NM_000059.4(*BRCA2*):c.5073dup ***	(p.Trp1692fs)	rs80359479	2 (1.4)	BC/OC	Pathogenic	Pathogenic (Reviewed by expert panel, guidelines-based)
NM_000059.4(*BRCA2*):c.6275_6276del	(p.Leu2092fs)	rs11571658	2 (1.4)	BC	Pathogenic	Pathogenic (Reviewed by expert panel, guidelines-based)
NM_000059.4(*BRCA2*):c.6415_6416delinsAT	(p.Glu2139Leu)	rs1064795067	1 (0.7)	BC	VUS **	Conflicting classifications of pathogenicity (criteria provided, single submitter, guidelines-based
NM_000059.3(*BRCA2*):c.9235delG	(p.Val3079fs)	rs397507422	1 (0.7)	BC	Pathogenic	Pathogenic (Reviewed by expert panel, guidelines-based)
NM_000059.3(*BRCA2*):c.9364G>C	(p.Ala3122Pro)	rs587782313	2 (1.4)	BC	Probably pathogenic	Conflicting classifications of pathogenicity (criteria provided, single submitter, guidelines-based)
NM_000059.4(*BRCA2*):c.9455A>G	(p.Glu3152Gly)	rs80359219	1 (0.7)	BC	VUS *	Conflicting classifications of pathogenicity (Uncertain significance (5); Likely benign (1)) (criteria provided, single submitter, guidelines-based)
NM_000059.4(*BRCA2*):c.9560A>G	(p.Asn3187Ser)	rs1329182873	1 (0.7)	BC	VUS **	Uncertain significance (multiple submitters, no conflicts, guidelines-based)
NM_000059.4(*BRCA2*):c.9812T>C	(p.Leu3271Ser)	----------------	1 (0.7)	BC	VUS **	Uncertain significance (multiple submitters, no conflicts, guidelines-based)
NM_000059.4(*BRCA2*):c.1838T>G	(p.Leu613Arg)	rs587780646	1 (0.7)	OC	VUS **	Conflicting classifications of pathogenicity (single submitter, guidelines-based)
NM_000059.4(*BRCA2*):c.3481_3482dup	(p.Asp1161fs)	rs878853569	1 (0.7)	OC	Pathogenic	Pathogenic (reviewed by expert panel, guidelines-based)
NM_000059.4(*BRCA2*):c.8839G>T	(p.Glu2947Ter)	rs398122715	1 (0.7)	OC	Pathogenic	Pathogenic (reviewed by expert panel, guidelines-based)

* From a total of 140 patients, ** variant of uncertain significance. *** One patient with BC and one with OC. **** ClinVar classifications indicate the aggregated clinical significance of each variant, while the review status reflects the level of consensus and adherence to established guidelines (e.g., ACMG/AMP). Terms such as Pathogenic, Uncertain significance, and Conflicting classifications of pathogenicity describe the overall interpretation, whereas qualifiers like reviewed by expert panel, multiple submitters, no conflicts, or single submitter indicate the strength and agreement of the evidence. The term guidelines-based denotes that the interpretation followed standardized classification criteria.

**Table 4:** Summary of Clinical and Pathological Characteristics by *BRCA2* Variant Status in Breast and Ovarian Cancer Patients.

Variable		Breast Cancer			Ovary Cancer		
		Variants					
		positive (*n* = 14)	negative (*n* = 102)	*p*-value	positive (*n* = 4)	negative (*n* = 15)	*p*-value
Age (years)							
	media ± SD	43.4 ± 13.1	47.49 ± 13.3	0.305	52.0 ± 10.9	56.50 ± 12.86	0.908
	Range	30–62	23–79		34–62	35–77	
BMI (Body Mass Index) kg/m^2^							
	Overweight 25.1 ± 30 (n, %)	4 (29)	6 (6)	0.019	2 (50)	2 (13)	0.171
Menopause Status							
	Yes (n. %)	2 (14)	15 (15)	1.0	0 (0)	12 (80)	0.018
	No (n. %)	12 (86)	87 (75)		4 (100)	3 (20)	
Family History of Cancer							
	First or second degree relative with breast, ovarian, or pancreatic cancer (n. %)	14(100)	58 (57)	0.004	2 (50)	9 (60)	1.0
Chemotherapy response							
	Complete	5 (36)	70 (69)	0.032	1 (25)	3 (21)	1.0

**Table 5:** *BRCA2* variant in women with BC and OC from Jalisco population.

HGVS (cDNA)	Protein	rsID	Frequency in This Study	Frequency in Other Studies/Populations (BC/OC)	Frequency in dbSNP (gnomAD **)	*p* Mexico vs. Other Study	*p* Mexico vs. dbSNP
c.9364G>C	(p.Ala3122Pro)	rs587782313	1.4 (2/140)	Portugal, OC: 1.05% (1/95) [30]	0.0000464 (65/1,401,414)	1.0	0.0
c.3264dup	(p.Gln1089fs)	rs80359380	2.1 (3/140)	Spain, BC: 8.47 (10/118) [31]/Mexico, TNBC *: 0.26 (1/387) [32].	0.000012 (7/573,348)	0.024 (Spain)/0.079 (Mexico)	0.0
c.8839G>T	(p.Glu2947Ter)	rs398122715	0.7 (1/140)	Not reported	0.00001 (1/78,578)		0.0036
c.1838T>G	(p.Leu613Arg)	rs587780646	0.7 (1/140)	Mexico, BC: 1.9 (1/51) [16]	0.0000129 (18/1,395,472)	0.4638	0.0019
c.9560A>G	(p.Asn3187Ser)	rs1329182873	0.7 (1/140)	Not reported	0.000003 (2/595,622)		0.0007
c.9812T>C	(p.Leu3271Ser)		0.7 (1/140)	Not reported	0.000003 (2/595,664)		0.0007
c.9235delG	(p.Val3079fs)	rs397507422	0.7 (1/140)	Mexico, BC and OC: 1.08 (1/92) [17]	0.0000050 (7/1,400,962)	1.0	0.0008
c.3481_3482dup	(p.Asp1161fs)		0.7 (1/140)	Brazil, BC: 8.3 (1/12) [33]	0.000002 (1/595,546)	0.1521	0.0005
c.5073dup	(p.Trp1692fs)	rs80359479	0.7 (1/140)	Brazil, BC: 0.4 (1/248) [34]	0.000023 (6/264,690)	1.0	0.0037
c.3188A>G	(p.Gln1063Arg)	rs775030825	0.7 (1/140)	Not reported	0.000005 (3/590,348)		0.0009
c.6275_6276del	(p.Leu2092fs)	rs11571658	1.4 (2/140)	Canada, OC: 0.000007 (1/1342) [35]/Mexico, BC: 0.026 (1/3842) [36]	0.0000753 (105/1,394,404)	0.0249 (Canada)/0.00046 (Mexico)	0.0001
c.6415_6416delinsAT	(p.Glu2139Leu)	rs1064795067	0.7 (1/140)	Not reported	Not reported		
c.9455A>G	(p.Glu3152Gly)	rs80359219	0.7 (1/140)	Not reported	0.000013 (8/595,596)		0.0021

* TNBC: Triple Negative Breast Cancer. ** Reported global frequencies including all the available ancestries in gnomAD.

**Table 6:** Functional and Clinical Annotations for *BRCA2* Variants (VEP Analysis).

Variant	Allele	Consequence	Exon	SIFT	PolyPhen	Clinical Significance
rs587780646	G	Missense	10	Deleterious (0.01)	Benign (0.357)	Uncertain Significance Likely Benign
rs775030825	G	Missense	11	Deleterious (0.02)	Benign (0.35)	Uncertain Significance Likely Benign
rs80359380	TT	Frameshift	11	-	-	Pathogenic
rs80359479	AAAAAAAA	Frameshift	11	-	-	Uncertain Significance Pathogenic
rs11571658	-	Frameshift	11	-	-	Pathogenic Likely Pathogenic
rs1064795067	AT	Missense	11	Deleterious (0)	Possibly damaging (0.738)	Uncertain Significance Likely Benign
rs398122715	T	Stop gained	22	-	-	Pathogenic
rs397507422	-	Frameshift	24	-	-	Pathogenic Likely Pathogenic
rs587782313	C	Missense	25	Deleterious (0)	Probably damaging (0.936)	Uncertain Significance Likely Pathogenic
rs80359219	G	Missense	25	Deleterious (0.02)	Benign (0.027)	Uncertain Significance
rs1329182873	G	Missense	26	Tolerated (0.22)	Benign (0.007)	Uncertain Significance

**Table 7:** Structural Consequences of Missense Variants in *BRCA2* Predicted by HOPE.

Variant (AA)	rsID	Key Changes (HOPE) *	HOPE Interpretation
(p.Leu613Arg)	rs587780646	↑ Size, Neutral → Positive, ↓ Hydrophobicity	Probably damaging
(p.Asn3187Ser)	rs1329182873	↓ Size, Negative → Neutral, ↑ Hydrophobicity	Probably damaging
(p.Gln1063Arg)	rs775030825	↑ Size, Neutral → Positive. Common substitution in other species	Probably not damaging
(p.Ala3122Pro)	rs587782313	↑ Size. Proline may induce structural changes (rigidity, helix disruption)	Probably damaging
(p.Leu3271Ser)	c.9812T>C	↓ Size, ↓ Hydrophobicity → loss of hydrophobic interactions	Probably damaging
(p.Glu2139Leu)	rs1064795067	↓ Size, Negative → Neutral, ↑ Hydrophobicity. Loss of charge, possible salt bridge disruption	Probably damaging
(p.Glu3152Gly)	rs80359219	↓ Size, Negative → Neutral, ↑ Flexibility. Loss of side chain interactions	Probably damaging

* Arrows indicate changes in amino acid properties: ↑ or ↓ Size denotes whether the mutant residue is larger or smaller than the wild-type; Charge shift refers to a change in the residue’s electrical charge (e.g., Neutral → Positive); Hydrophobicity indicates a change in the residue’s affinity for hydrophobic environments, where ↑ means more hydrophobic and ↓ means less hydrophobic than the wild-type residue.

**Table 8:** Integrated summary and suggested priority level of *BRCA2* variants based on domain localization, computational predictions and clinical classification.

Variant (cDNA)	Protein Change	rsID	Functional Domain (UniProt)	Variant Type	SIFT	PolyPhen	HOPE Summary	ClinVar Classification	Suggested Priority Level *
c.3264dup	p.Gln1089fs	rs80359380	BRC repeats (1003–2082)	Frameshift	NA	NA	Truncating, loss of downstream BRC repeats	Pathogenic, expert panel	High
c.5073dup	p.Trp1692fs	rs80359479	BRC repeats (1003–2082)	Frameshift	NA	NA	Truncating	Pathogenic, expert panel	High
c.3481_3482dup	p.Asp1161fs	rs878853569	BRC repeats (1003–2082)	Frameshift	NA	NA	Truncating	Pathogenic, expert panel	High
c.8839G>T	p.Glu2947Ter	rs398122715	DNA-binding domain (2804–3054)	Stop gained	NA	NA	Premature stop	Pathogenic, expert panel	High
c.9235delG	p.Val3079fs	rs397507422	Terminal DBD (3052–3185)	Frameshift	NA	NA	Truncating	Pathogenic/Likely pathogenic	High
c.6275_6276del	p.Leu2092fs	rs11571658	Outside described domains	Frameshift	NA	NA	Truncating	Pathogenic, expert panel	High
c.9364G>C	p.Ala3122Pro	rs587782313	Terminal DBD (3052–3185)	Missense	Deleterious (0.00)	Probably damaging (0.936)	Proline may induce rigidity and helix disruption	Conflicting: VUS/Likely pathogenic	High
c.9455A>G	p.Glu3152Gly	rs80359219	Terminal DBD (3052–3185)	Missense	Deleterious (0.02)	Benign (0.027)	Loss of side-chain interactions and ↑ flexibility	Uncertain significance	Moderate
c.6415_6416delinsAT	p.Glu2139Leu	rs1064795067	Outside described domains	Missense	Deleterious (0.00)	Possibly damaging (0.738)	Loss of negative charge, possible salt bridge disruption	Conflicting, single submitter	Moderate
c.1838T>G	p.Leu613Arg	rs587780646	Outside described domains	Missense	Deleterious (0.01)	Benign (0.357)	↑ size, neutral → positive charge, ↓ hydrophobicity	VUS/Likely benign	Moderate
c.9560A>G	p.Asn3187Ser	rs1329182873	C-terminal region/DBD boundary	Missense	Tolerated (0.22)	Benign (0.007)	↓ size, charge shift, ↑ hydrophobicity	Uncertain significance	Low
c.9812T>C	p.Leu3271Ser	—	Outside described domains	Missense	Not in VEP	Not in VEP	↓ size and hydrophobicity, loss of hydrophobic interactions	Uncertain significance	Low
c.3188A>G	p.Gln1063Arg	rs775030825	BRC repeats (1003–2082)	Missense	Deleterious (0.02)	Benign (0.35)	Larger Arg; HOPE “probably not damaging”	VUS/Likely benign	Moderate

* Note: Suggested priority levels were determined following a stepwise rubric. Truncating variants (frameshift or stop-gained) were directly classified as High. For missense variants, localization within functionally critical BRCA2 domains (BRC repeats, DNA-binding domain) increased the priority by one level. Concordant deleterious predictions from both SIFT (≤0.05) and PolyPhen (≥0.85) increased the priority by one level; if only one predictor indicated damage, the maximum classification was Moderate. ClinVar entries classified as pathogenic or likely pathogenic increased the priority by one level, with High as the upper limit. HOPE structural predictions were used to support interpretation but did not independently raise the assigned priority. This prioritization is proposed solely as a research guide and does not constitute definitive pathogenicity evidence. Arrows indicate changes in amino acid properties: ↑ or ↓ Size denotes whether the mutant residue is larger or smaller than the wild-type; Charge shift refers to a change in the residue’s electrical charge (e.g., Neutral → Positive); Hydrophobicity indicates a change in the residue’s affinity for hydrophobic environments, where ↑ means more hydrophobic and ↓ means less hydrophobic than the wild-type residue.

## Data Availability

The original contributions presented in this study are included in the article. Further inquiries can be directed to the corresponding author.

## Supplementary Materials

The following supporting information can be downloaded at https://www.mdpi.com/article/10.3390/medsci13040248/s1, Table S1: Comparison of Clinical and Pathological Characteristics by BRCA2 Variant Status in Breast and Ovarian Cancer Patients.; Table S2: Structural Localization of BRCA2 Variants within Functional Domains and Their Associated Roles.
