# Supplementary material for: Identification and Computational Analysis of BRCA2 Variants in Mexican Women from Jalisco, Mexico, with Breast and Ovarian Cancer
Source: Med Sci (Basel). 2025 Oct 29;13(4):248. doi: 10.3390/medsci13040248 (PMC12641962; doi:10.3390/medsci13040248)
Supplement: Supplementary file 1 [file medsci-13-00248-s001.zip › medsci-3783058-supplementary.pdf]

**Table S1.** Comparison of Clinical and Pathological Characteristics by *BRCA2* Variant Status in Breast and Ovarian Cancer Patients.

| **Variable** | | BC | | | OC | | |
|---|---|---|---|---|---|---|---|
| | | ***BRCA2* status** | | | | | |
| | | positive (n=14) | negative (n=102) | p-value | positive (n=4) | negative (n=15) | p-value |
| **Age (years)*** | | | | | | | |
| | media ± SD** | 43.4 ± 13.1 | 47.49 ± 13.3 | 0.305 | 52.0 ± 10.9 | 56.50 ± 12.86 | 0.908 |
| | Range | 30–62 | 23-79 | | 34-62 | 35-77 | |
| **Age at Menarche (years)** | | | | | | | |
| | media ± SD* | 11.5 ± 1.19 | 12.36 ± 1.55 | 0.068 | 12.2 ± 0.83 | 11.94± 0.89 | 0.758 |
| | Range | 9–14 | 8-16 | | 11-13 | 10-13 | |
| **BMI (Body Mass Index) kg/m$^2$** | | | | | | | |
| | Normal 18.5 ± 25 (n, %) | 8 (57) | 81 (79) | 0.130 | ------- | 10 (66) | 0.121 |
| | Overweight 25.1 ± 30 (n, %) | 4 (29) | 6 (6) | **0.019** | 2 (50) | 2 (13) | 0.171 |
| | Obese >31.9 (n. %) | 2 (14) | 15 (15) | 1.0 | 2 (50) | 3 (21) | 0.272 |
| **Alcohol Consumption** | | | | | | | |
| | Yes (n. %) | 2 (14) | 16 (16) | 1.0 | 1 (25) | 3 (20) | 1.0 |
| | No (n. %) | 12 (86) | 86 (84) | | 3 (75) | 12 (80) | |
| **Tobacco Consumption** | | | | | | | |
| | Yes (n. %) | 2 (14) | 11 (11) | 1.0 | 1 (25) | 1 (7) | 0.385 |
| | No (n. %) | 12 (86) | 91 (89) | | 3 (75) | 14 (93) | |
| **Hormonal Consumption** | | | | | | | |
| | Yes (n. %) | 5 (36) | 48 (47) | 0.607 | 0 (0) | 0 (0) | 0.480 |
| | No (n. %) | 9 (64) | 54 (53) | | 4 (100) | 15 (100) | |
| **Menopause Status** | | | | | | | |
| | Pre-menopause (n. %) | 5 (36) | 54 (53) | 0.355 | 0 (0) | 1 (7) | 1.0 |
| | menopause (n. %) | 9 (64) | 48 (47) | | 4 (100) | 14 (93) | |
| **Abortion** | | | | | | | |
| | Yes (n. %) | 2 (14) | 15 (15) | 1.0 | 0 (0) | 12 (80) | **0.018** |
| | No (n. %) | 12 (86) | 87 (75) | | 4 (100) | 3 (20) | |
| **Breastfeeding** | | | | | | | |
| | Yes (n. %) | 9 (64) | 73 (72) | 0.803 | 4 (100) | 10 (67) | 0.621 |
| | No (n. %) | 5 (36) | 29 (28) | | 0 (0) | 5 (33) | |
| **Family History of Cancer** | | | | | | | |
| | First or second degree relative with breast, ovarian, or pancreatic cancer (n. %) | 14(100) | 58 (57) | **0.004** | 2 (50) | 9 (60) | 1.0 |
| | First or second degree relative with other cancer type (n. %) | 0 (0) | 20 (20) | 0.299 | 1 (25) | 2 (13) | 0.530 |
| | No (n. %) | 0 (0) | 24 (23) | 0-189 | 1 (25) | 4 (27) | 1.0 |
| **Detection cancer** | | | | | | | |
| | Autodetection (n. %) | 13 (93) | 95 (93) | 1.0 | 0 (0) | 0 (0) | 1.0 |
| | Ultrasonogram/ecosonogram (n. %) | 1 (7) | 9 (7) | | 4 (100) | 15(100) | 1.0 |
| **Disgostic years** | | | | | | | |
| | 1-4 (n, %) | 9 (65) | 72 (71) | 1.0 | 4 (100) | 9 (60) | 0.368 |

| | | | | | | |
|---|---|---|---|---|---|---|
| 5-9 (n, %) | 3 (21) | 14 (15) | 0.429 | 0 (0) | 6 (40) | 0.612 |
| 10 and more (n, %) | 2 (14) | 16 (16) | 1.0 | 0 (0) | 0 (0) | 0.106 |
| **Laterality** | | | | | | |
| Unilateral (n, %) | 12 (86) | 94 (92) | 0.345 | 1 (25) | 13 (87) | **0.037** |
| Bilateral (n, %) | 2 (14) | 8 (8) | | 3 (75) | 2 (13) | |
| **Clinical Stage** | | | | | | |
| In situ | 1 (7) | 2 (2) | 0.109 | 0 (0) | 0 (0) | 1.0 |
| I | 3 (21) | 17 (17) | 0.699 | 0 (0) | 1 (6) | 1.0 |
| II | 5 (36) | 37 (36) | 1.0 | 0 (0) | 1 (6) | 1.0 |
| III | 5 (36) | 35 (34) | 1.0 | 3 (75) | 6 (40) | 0.303 |
| IV | 0 (0) | 11(11) | 0.690 | 1 (25) | 7(48) | 0.602 |
| **Histology** | | | | | | |
| Ductal | 14 (100) | 95 (93) | 1.0 | 0 (0) | 0 (0) | 1.0 |
| Lobular | 0 (0) | 6 (6) | 1.0 | 0 (0) | 0 (0) | 1,0 |
| Mix | 0 (0) | 1 (1) | 0.354 | 0 (0) | 0 (0) | 1.0 |
| High-grade serous | 0 (0) | 0 (0) | 1.0 | 4 (100) | 15 (100) | 1.0 |
| **Molecular subtype** | | | | | | |
| Luminal A | 2 (14) | 19 (19) | 1.0 | 0 (0) | 0 (0) | 1.0 |
| Luminal B | 3 (21) | 30 (29) | 0.754 | 0 (0) | 0 (0) | 1.0 |
| Triple negative | 7 (51) | 50 (49) | 1-0 | 0 (0) | 0 (0) | 1.0 |
| Luminal A/ B | 1 (7) | 1(1) | 0.571 | 0 (0) | 0 (0) | 1.0 |
| Triple negative/Luminal A | 1 (7) | 2 (2) | 0.322 | 0 (0) | 0 (0) | 1.0 |
| High-grade serous | 0 (0) | 0 (0) | 1.0 | 4 (100) | 15 (100) | 1.0 |
| **KI-67** | | | | | | |
| < 20% | 3 (21) | 19 (19) | 1.0 | 0 (0) | 0 (0) | 1-0 |
| ≥ 20% | 11 (79) | 83 (81) | 1.0 | 4 (100) | 15 (100) | 1.0 |
| **Lymph Node** | | | | | | |
| positive | 4 (29) | 27 (26) | 1.0 | 1 (25) | 4 (27) | 1.0 |
| negative | 10 (71) | 75 (74) | 1.0 | 3 (75) | 11 (73) | 1.0 |
| **Chemotherapy response** | | | | | | |
| Complete | 5 (36) | 70 (69) | **0.032** | 1 (25) | 3 (21) | 1.0 |
| Partial | 6 (43) | 27 (26) | 0.356 | 1 (25) | 8 (53) | 0.582 |
| No response | 2 (14) | 2 (2) | 0.070 | 0 (0) | 2 (13) | 1-0 |
| Recurrency | 1 (7) | 3 (3) | 0.405 | 2 (50) | 2 (13) | 0.178 |

**Table S2.** Structural Localization of BRCA2 Variants within Functional Domains and Their Associated Roles.

| Variant | Protein change (AA) | Functional domain | Domain range | Domain function |
|---|---|---|---|---|
| rs398122715 | p.Glu2947Ter | DBD (DNA-binding domain) | 2804–3054 | Binds single- and double-stranded DNA and interacts with DSS1 for homologous recombination repair. |
| rs80359380 | p.Gln1089fs | BRC repeats | 1003–2082 | Binds RAD51 to promote nucleofilament formation in homologous recombination. |
| rs587780646 | p.Leu613Arg | – | – | Outside known functional domains. |
| rs1329182873 | p.Asn3187Ser | C-terminal region / DBD boundary | 3190–3418 | Involved in phosphorylation, nuclear localization, and RAD51 interaction. |
| rs775030825 | p.Gln1063Arg | BRC repeats | 1003–2082 | Binds RAD51 for homologous DNA repair. |
| rs587782313 | p.Ala3122Pro | DBD terminal | 3052–3185 | Binds DNA and DSS1; contributes to structural architecture of the domain. |
| c.3481_3482dup | p.Asp1161fs | BRC repeats | 1003–2082 | Binds RAD51 to stabilize DNA repair nucleofilaments. |
| rs80359479 | p.Trp1692fs | BRC repeats | 1003–2082 | Binds RAD51 for homologous recombination. |
| — | — | EPITOPE QTSLLAKKW (ID 600651) | 1683–1692 | — |
| **c.9812T>C** | p.Leu3271Ser | C-terminal region / DBD boundary | 3190–3418 | Involved in phosphorylation, nuclear localization, and RAD51 interaction. |
| rs397507422 | p.Val3079fs | DBD terminal | 3052–3185 | Binds DNA and DSS1; contributes to structural architecture of the domain. |
| rs11571658 | p.Leu2092fs | – | – | Outside known functional domains. |
| **c.6415_6416delinsAT** | p.Glu2139Leu | – | – | Outside known functional domains. |
| c.9455A>G | p.Glu3152Gly | DBD terminal | 3052–3185 | Binds DNA and DSS1; contributes to structural architecture of the domain. |
